# Feasibility of lattice radiation therapy using a noncoplanar vertex arrangement and dynamic conformal arcs

**DOI:** 10.1002/acm2.70818

**Published:** 2026-09-23

**Authors:** Haixia Cui, Wei Huang, Xin Yi, Wenli Lu, Qingfeng Jiang

**Affiliations:** ^1^ Department of Oncology Key Laboratory of Immunity Inflammation & Cancer (Chongqing Municipal Health Commission) The First Affiliated Hospital of Chongqing Medical University Chongqing China

**Keywords:** couch rotation limit, DCA planning, LRT, non‐coplanar vertex arrangement

## Abstract

**Background:**

Current Lattice radiation therapy (LRT) mainly uses inverse IMRT/VMAT optimization, which is time‐consuming and complex; however the high modulation complexity compromises dosimetric accuracy and blurs the peak‑to‑valley dose ratio.

**Purpose:**

This study presents a simple noncoplanar vertex arrangement for dynamic conformal arc (DCA)‑based LRT and evaluates its feasibility.

**Methods:**

Different noncoplanar vertex geometries were generated in a water phantom, and DCA‑based LRT plans were designed using Eclipse v15.6 (Varian). The LRT target volume (LTV) was the union of all vertices. Single‑fraction 20‑Gy plans were generated with three coplanar 6‑MV FFF beams and calculated with AcurosXB calculation. Optimal vertex geometry was selected based on high‑dose proportion (measured with the volume ratio of prescribed dose structure to GTV [V_Dp_ / V_GTV_], and the volume ratio of LTV to GTV [V_LTV_ / V_GTV_]), GTV dose metrics, heterogeneity(measured with peak‐to‐valley‐dose‐ratio [PVDR], Volume Dose Ratio [VDR] and other three dose ratios defined as Dose matrix of ‘peak’ / ‘valley’ [Dp / D_95% Gap_, D_mean LTV_ / D_mean Gap_, and D_2cc GTV_/ D_95% Gap_]), valley dose (D_95% Gap_ and D_5% Gap_) and normal tissue(NT) max dose, dose profiles/isodose distributions and plan complexity (MCS, SAS, MU/cGy). The same strategy was applied to two gastrointestinal stromal tumor (GIST) patients with evaluation of OAR sparing and planning time.

**Results:**

In the phantom, six noncoplanar vertex configurations and one coplanar vertex configuration were generated, and corresponding DCA plans were designed for each. All plans achieved a high‑dose volume proportion exceeding 1% and EUD_GTV_ of 5.07 Gy ‐ 6.78 Gy, with high dose heterogeneity (high PVDR, Dp / D_95% Gap_, D_mean V_ / D_mean Gap_ and moderate VDR). Plan complexity remained low across all configurations, with MCS ranged 0.68–0.79. Optimal vertex geometry was 1‐cm diameter with a 4‐cm center‑to‑center distance, providing a favorable balance between dose heterogeneity and valley dose sparing. Moreover, the DCA plan based on noncoplanar vertex arrangement yielded dose distributions comparable to those of the non‐coplanar plan with couch rotation. Importantly, both normal tissue (NT) doses in all phantom plans were very low. The clinical applicability was verified in two patients, with all dosimetric parameters meeting the recommended LRT ranges. Consistent with the phantom findings, patient NT and OAR doses remained far below the SBRT constraints, with a total planning time of ∼1 hour per patient, supporting the safety and translational potential of this approach.

**Conclusions:**

DCA‑based LRT with noncoplanar vertices is feasible on linacs, simplifying planning and enabling accurate delivery. It offers a valuable alternative method for patients with couch rotation limit. Optimal vertex arrangement with 1‑cm diameter and 3.5–4 cm center to center distance obtained in water phantom. Two patient cases met LRT criteria, with a total planning time of approximately one hour per patient, supporting clinical applicability in complex anatomies.

## 1 INTRODUCTION

Spatially fractionated radiation therapy (SFRT) delivers separated high radiation doses to the scattered vertices and low doses to the intervening gaps, creating a highly heterogeneous dose distribution within the tumor while minimizing toxicity to surrounding tissues.^1^ Previous studies^2^, ^3^ have shown the good clinical effects, and SFRT is now widely accepted as a valuable alternative for the management of bulky and locally advanced tumors.^4^, ^5^ Its enhanced antitumor efficacy is thought to be attributed to the heterogeneous dose pattern: the high‑dose “peaks” function similarly to stereotactic radiation therapy delivered to discrete tumor sub‑volumes, while the low‑dose “valleys” help preserve the tumor microenvironment and vasculature—potentially promoting bystander and abscopal effects that may contribute to the improved tumor response.^2^, ^6^, ^7^, ^8^ Consequently, parameters characterizing dose heterogeneity (e.g., peak‐to‐valley‐dose‐ratio (PVDR), Volume Dose Ratio (VDR)) are considered key for SFRT plan evaluation.^9^ In addition, several studies have identified valley dose as the factor most strongly correlated with overall tumor response.^5^, ^10^, ^11^, ^12^

Since Wu et al.^13^ proposed an extension of 2D GRID therapy into modern 3D high‑dose LATTICE radiotherapy (LRT) in 2010, several methods have been applied to SFRT delivery.^4^, ^14^ MLC‑based LRT offers greater flexibility in lattice definition and plan optimization than GRID techniques, enabling more conformal dose distributions and better OAR sparing.^9^ Current LRT predominantly relies on inverse IMRT/VMAT optimization, which is time‑consuming and complex^4^, ^14^, ^15^, ^16^, ^17^; even automatic planning takes dozens of minutes on average.^17^ Moreover, increased modulation complexity and leaf motion degrade dosimetric accuracy and blur the peak‐to‐valley dose ratio.^18^ Dynamic conformal arc (DCA) therapy has been widely used in SBRT due to its high efficiency and robustness^19^, ^20^, ^21^—attributes that align well with SFRT requirements. DCA can reduce the interplay‑induced dose ambiguity and greatly shorten planning time—a major barrier to clinical adoption. We therefore applied DCA to LRT to simplify the plan design process. However, the main challenge is that two or more vertices lie on the same transverse plane; consequently, MLC leaf pairs cannot conform precisely to all targets across all gantry angles, causing unintended irradiation between adjacent vertices and elevating valley dose. Thus, vertex arrangement is important and remains one of the major challenges in the clinical implementation of LRT. Manual vertex placement in 3D space while maintaining specific inter‑vertex distances is laborious and difficult within TPS.^4^, ^22^, ^23^ Some centers have developed in‑house programs for automatic vertex placement,^15^, ^16^, ^24^, ^25^ and others have integrated vertex position optimization into dose optimization.^26^, ^27^ However, these approaches have high entry barriers and are generally not applicable to DCA plans, limiting their comprehensive clinical application.

Here, we propose a simple and practical noncoplanar vertex arrangement method. By applying a proper coronal‑plane rotation, the complex 3D vertex arrangement is transformed into a quasi‐ 2D configuration, greatly simplifying the placement process and enabling DCA‑based LRT planning with good applicability, efficiency, and robustness.

## 2 MATERIALS AND METHODS

Treatment planning was performed using the Varian Eclipse treatment planning system (TPS, version 15.6, Varian Medical Systems, Palo Alto, CA). Dose calculations were conducted with the Acuros XB algorithm using a 1.0‑mm calculation grid. Both phantom and patient plans were generated with 6‑MV flattening‑filter‑free (FFF) photon beams. Target volumes were contoured, and dynamic conformal arc (DCA) plans were designed as described below. All plans were generated for delivery on a Varian VitalBeam linear accelerator equipped with a standard Millennium 120‑leaf MLC (central leaf width: 5 mm; outer leaf width: 10 mm).

### 2.1 Dynamic conformal arc plan for non‐coplanar vertices

#### 2.1.1 Arrangement of non‐coplanar vertices

**FIGURE 1:**
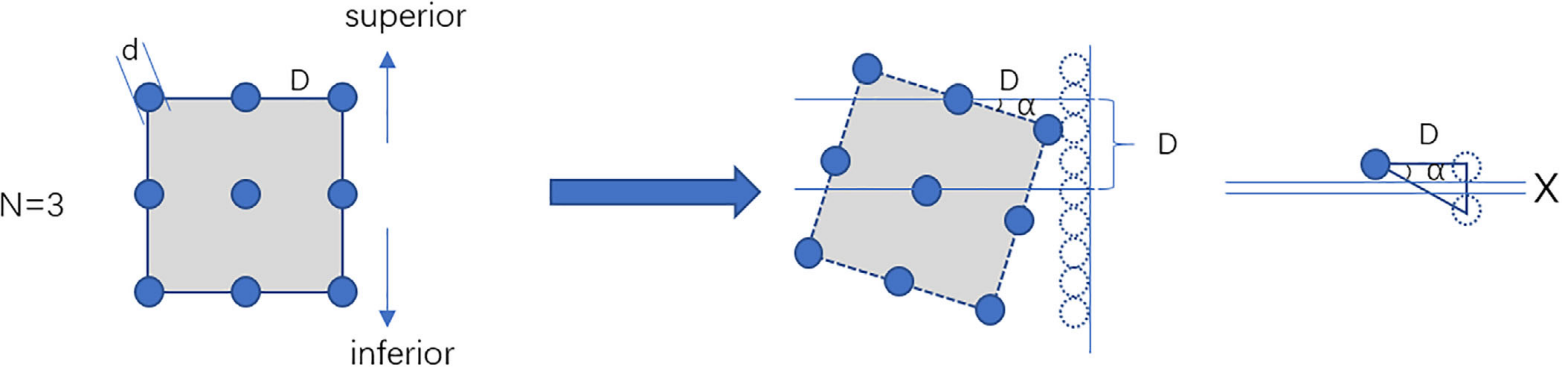
Geometric representation of non‐coplanar rotation and vertex arrangement. Blue dots represent vertices of one group (diameter: d cm). No rotation(left), Coronal‑plane rotation with angle 𝛼 (middle), Approximate calculation of angle 𝛼 (right). Vertices are separated by a center‑to‑center distance of D cm. Three vertices in one group on the same slice (𝑁 = 3).

The non‐coplanar vertices were organized into columns and distributed using a rotational strategy in either the coronal (used here) or the sagittal plane. To generate a 3D noncoplanar lattice, an initial rotation was applied in the coronal plane. The rotation angle (𝛼) was determined mathematically from the number of vertices (𝑁) in each group on the same axial slice. The geometric relationship is given by Equation 1:

(1) $$\tan\alpha =\;\;\frac{d+x}{D}=\frac{d+x}{\left(d+x\right)\cdot N}=\frac{1}{N}\;$$

where 𝛼 is the rotation angle and 𝑁 is the number of vertices in a group on the same slice. For example, if a group contains three vertices on a single slice, the rotation angle is calculated as 𝛼 = arctan (1/3). A representative schematic is shown in Figure 1.

**FIGURE 2:**
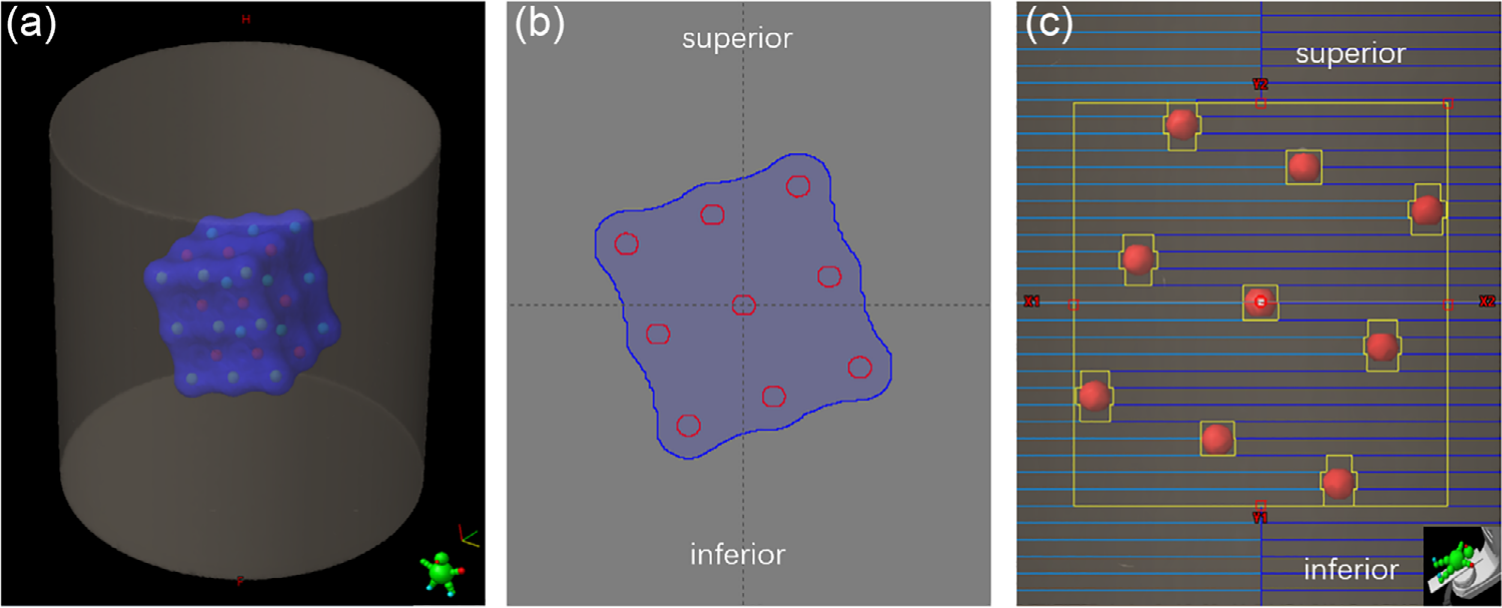
An example of 3 × 3 × 3 non‐coplanar vertex arrangement with diameter = 1 cm (d1) and center‐to‐center distance = 4 cm (D4).

Furthermore, during the DCA delivery, the MLC apertures were dynamically shaped at each control point. As shown in the beam's‐eye view (BEV) in Figure 2c, the MLCs were configured to tightly conform to the target vertices while blocking the inter‐vertex regions in the superior‐inferior direction.

Prior to vertex delineation, the coronal images were rotated by the specific angle α (determined by N). Vertices were then contoured on the transverse planes. The initial vertex was placed at the geometric center of the gross target volume (GTV) using the 3D brush tool in the TPS contouring interface. A user‐defined grid guide was overlaid on the image, and subsequent vertices were placed at the grid intersections along the left‐right direction with a spacing of D inside GTV. This process was repeated along the superior‐inferior direction by shifting the distance of D until it extended beyond the GTV boundary. The contoured vertices were saved as the first group (G1). Additional groups (e.g., G2, and G3) were generated by translating the G1 structure by D cm in the ventral or dorsal direction using the Transform Structure tool. The vertex group with a 1‑cm diameter and 4‑cm center‑to‑center distance was designated as D4d1G1, where D = 4 cm, d = 1 cm, and G1 denotes Group 1.

To ensure safe dose fall‐off and spare adjacent healthy tissues, all vertices were confined within a 1‐cm inner margin from the GTV. The LRT target volume (LTV) was then generated as the union of all vertices. The normal tissue (NT) structure was defined as the body minus the GTV, and was longitudinally limited to ± 10 cm superior and inferior to the GTV.

#### 2.1.2 DCA LRT planning

For each LTV, DCA plans were established using three dynamic SRS ARC beams without couch rotation. The gantry rotated from 178° to 182° (or vice versa) using 6‐MV FFF beams at a dose rate of 1400 MU/min. The isocenter was placed at the geometric center of the LTV. MLC apertures were dynamically shaped to conform tightly to each vertex group without any additional margin. Three fields (field 1, field 2 and field 3) were assigned to irradiate the corresponding vertex groups (D4d1G1, D4d1G2, and D4d1G3), respectively. Volume dose was calculated, and beam MUs were adjusted to ensure that ≥95% of each vertex group received the prescribed 20‐Gy dose (e.g., the Mus for field 1 were set to cover 95% of D4d1G1). Ultimately, these DCA plans were delivered highly concentrated doses to the LTV while minimizing dose to non‐LTV regions within the GTV, thus creating a spatially heterogenic dose distribution.

### 2.2 Ideal phantom parameter optimization

#### 2.2.1 Experimental setup

A cylindrical water phantom with a diameter 30 cm and length 30 cm was created. A 3×3×3 vertex arrangement was designed within the phantom with a rotation angle of 19° (tan19°≈ 1/3). Multiple vertex configurations with varying diameters and center‑to‑center distances were evaluated (e.g., D3d1, D3.5d1, D3.5d1.4, D4d1, D4d1.4, D5d1). The D4d1.4 configuration was derived from D4d1 by applying a 0.2‐cm isotropic margin in the axial plane, excluding the superior‐inferior direction. Since no anatomical target was present, a virtual GTV was generated based on the LTVs (4‑cm expansion followed by 3‑cm inward contraction) to create a perfectly matched geometric reference for parameter optimization. An example of 3×3×3 non‐coplanar vertex arrangement is shown in Figure 2.

#### 2.2.2 Plan evaluation and parameter selection

To determine the optimal parameters, we evaluated both geometric and dosimetric parameters in the ideal phantom. The selection of these metrics was based on established practices in LRT dosimetric evaluation and is detailed as follows^9^, ^17^:

The high‐dose proportion was quantified by the ratios of V_Dp_ / V_GTV_ and V_LTV_ / V_GTV_, where V_Dp_ represents the volume receiving the prescribed dose. A higher ratio indicates a larger high‐dose area. Target dose metrics included the dose covering X% of GTV (D_X%GTV_, with X = 5%, 10%, 50%, and 90%), the dose covering 2 cm^3^ of GTV (D_2cc GTV_), the GTV mean dose (Dmean_GTV_), and equivalent uniform dose (EUD_GTV_). EUD was calculated according to the Equation 2, ^17^,

(2) $$\mathrm{EUD}=-\frac{\alpha }{2\beta }+\frac{1}{2}{\left[{\left(\frac{\alpha }{\beta }\right)}^{2}-\frac{4}{\beta }\ln\left(\sum_{j}v_{j}e^{-\alpha D_{j}-\beta {D_{j}}^{2}}\right)\right]}^{\frac{1}{2}}$$

where *D_j_* is the dose in the fractional volume *v_j_* and *α* = 0.38 Gy and β = 0.038 Gy.

The dose distribution between vertices was analyzed using the “Gap” structure, defined as the GTV excluding the LTV expanded by a 1‐cm margin. The mean dose, D95% and D5% of Gap were reported. Dose heterogeneity was quantified using the PVDR (PVDR = Dmax/Dmin within GTV), VDR (VDR = GTVD_10%_/GTVD_90%_), and three additional heterogeneity ratios: Dp / D_95% Gap_, D_mean LTV_ / D_mean Gap_, and D_2cc GTV_/ D_95% Gap_. Higher values for these metrics indicate greater dose heterogeneity. NT sparing was evaluated using the maximum dose and the volume receiving 50% of the prescribed dose (NT V50%). Dose distributions and dose profiles across multiple directions were compared after the coronal‑plane rotation.

Plan complexity was quantified using the modulation complexity score (MCS),^28^ the small aperture score (SAS)^29^ and the MU/cGy ratio. The MCS is a dimensionless index defined between 0 and 1, with a lower value indicating higher complexity. SAS measures the proportion of monitor units (MUs) delivered through small MLC apertures; a lower SAS value generally indicates simpler beam shapes and larger average apertures, which are associated with better dosimetric accuracy. The MU/cGy ratio was recorded as a surrogate for the treatment time.

### 2.3 Clinical examples

This retrospective study was approved by the ethics committee at our institution. All patient data were anonymized prior to analysis.

#### 2.3.1 Patient selection and plan design

After establishing the optimal phantom parameters, we evaluated the clinical dosimetric performance of the non‐coplanar arrangement in two patients with large (> 10 cm) gastrointestinal stromal tumor (GIST). A radiation oncologist contoured the GTV and OARs on 1‐mm CT slices. Using the method described in Section “Dynamic Conformal Arc plan for non‐coplanar vertices”, we arranged non‐coplanar vertices (d = 1 cm, D = 4 cm) and adapted their positions to the actual GTV shape, and designed DCA LRT plans.

#### 2.3.2 Clinical plan evaluation

For the two patient cases, the same dosimetric metrics as in the phantom study‐including high‐dose proportion, dose heterogeneity, GTV dose metrics, Gap and NT metrics, dose distributions and profiles, and plan complexity (MCS, SAS, and MU/cGy)‐ were evaluated to allow direct comparison. Vertex contouring time and planning time were also recorded to assess clinical workflow feasibility.

## 3 RESULTS

### 3.1 Dosimetric characteristics in ideal phantom

**FIGURE 3:**
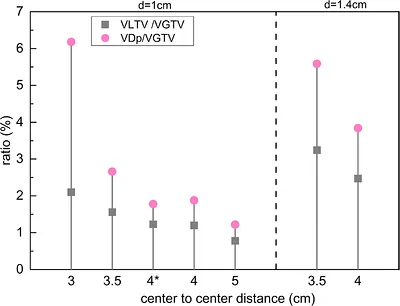
The high‐dose proportion parameters (VLTV/VGTV and VDp/VGTV) of each LTV. *denotes the plan for the 3 × 3 × 3 LTV D4d1* with 19° couch rotation.

**FIGURE 4:**
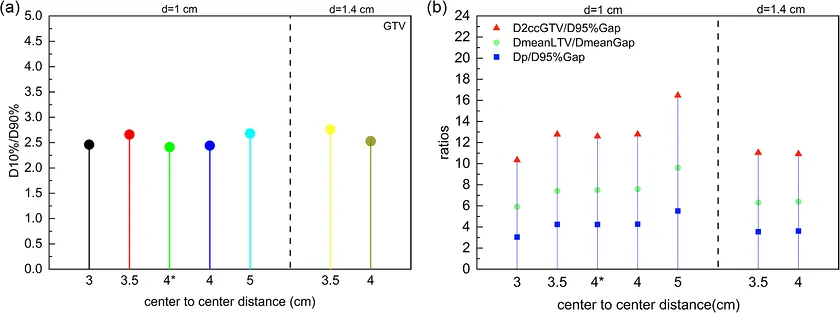
VDR (GTVD_10%_/GTVD_90%_) (a) and three additional dose inhomogeneity parameters (Dp / D_95% Gap_, D_mean LTV_ / D_mean Gap_, and D_2cc_ / D_95% Gap_) (b). *denotes the plan for the 3 × 3 × 3 LTV D4d1* with 19° couch rotation.

**FIGURE 5:**
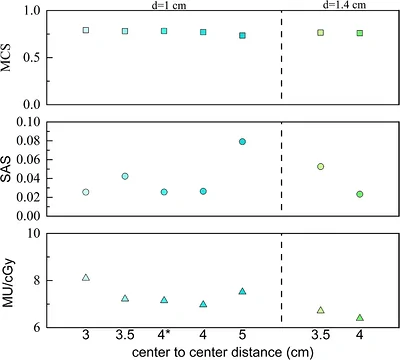
Modulation complexity score (MCS) (top), Small Aperture Score (SAS) (middle), and MU/cGy ratio (bottom) for each LTV considered in this study. *denotes the plan for the 3 × 3 × 3 LTV D4d1* with 19° couch rotation.

Based on different combinations of vertex diameter (d) and center‑to‑center distance (D), a total of six non‐coplanar LTVs and corresponding DCA plans were generated on a water phantom. One coplanar LTV without rotation and corresponding couch‑rotated plan (D4d1*) were also generated. For clarity, the plan for the 3×3×3 LTV D4d1* with 19° couch rotation are denoted with an asterisk (*) in the following figures. The critical dose‐volume parameters for all seven plans are presented in Figures 3, 4, 5, Figures S1–S3 and Table S1. All plans exhibited high PVDRs ranging from 22.22 to 31.54, and VDRs ranging from 2.41 to 2.68.

#### 3.1.1 High‐dose proportion parameters in different vertex arrangements

For each LTV, *V_Dp_/V_GTV_* (1.22%–6.18%) was consistently higher than *V_LTV_/V_GTV_* (0.78%–3.24%), with both parameters showing similar trends across different vertex arrangements (Figure 3). Both *V_LTV_/V_GTV_* and *V_Dp_/V_GTV_* decreased with increasing D in each fixed d. Conversely, at a fixed D, both ratios nearly doubled when d increased from 1 cm to 1.4 cm. D4d1 and D4d1* exhibited comparable values for both parameters.

#### 3.1.2 Dose inhomogeneity parameters in different vertex arrangements

As shown in Figure 4, the VDR (2.41–2.76) increased slightly with increasing vertex size (d) for each fixed D, but showed no clear trend with varying D for each fixed d. Conversely, Dp / D_95% Gap_ (3.05–5.52), D_mean LTV_ / D_mean Gap_ (2.76–4.08), and D_2cc_ / D_95% Gap_ (4.42–6.87) decreased with increasing d at a fixed D, and increased with increasing D at a fixed d, except for D_2cc_ / D_95% Gap_ at D = 3.5 cm.

#### 3.1.3 GTV, Gap, and NT dose metrics in different vertex arrangements

As shown in Figure S1, the D_meanGTV_ (32.9%–57.3%) and EUD_GTV_ (5.07–6.78 Gy) increased with increasing d for each fixed D, but decreased with increasing D at a fixed d, with EUD_GTV_ values comparable between D = 3.5 cm and D = 4.0 cm. The gap dose metrics (D_meanGap_, D_5%Gap_, D_95%Gap_) decreased with increasing D for each fixed d, and increased with increasing d at a fixed D (Figure S2). As shown in Figure S3, NT max dose (60.1%–76.6%) and V50% (4.05–49.05 cm^3^) followed similar trends to the gap metrics, except at D = 5 cm.

#### 3.1.4 Plan complexity in different vertex arrangements

As shown in Figure 5 (top), the MCS values (0.73–0.79) were similar across different LTVs. As shown in Figure 5 (middle), all plans yielded low SAS values (< 10%, 2.33%–7.91%). The SAS remained relatively consistent for each D at d = 1.0 cm, except for D = 5.0 cm, and showed minimal variation with varying d at a fixed D. The MU/cGy ratio (6.39–8.10) decreased with increasing D at d = 1 cm, and similarly decreased with increasing d at a fixed D (Figure 5, bottom). These findings collectively indicate that the proposed DCA‑based LRT plans exhibited consistently low plan complexity across all evaluated configurations.

#### 3.1.5 Dose distributions and profiles in different vertex arrangements

**FIGURE 6:**
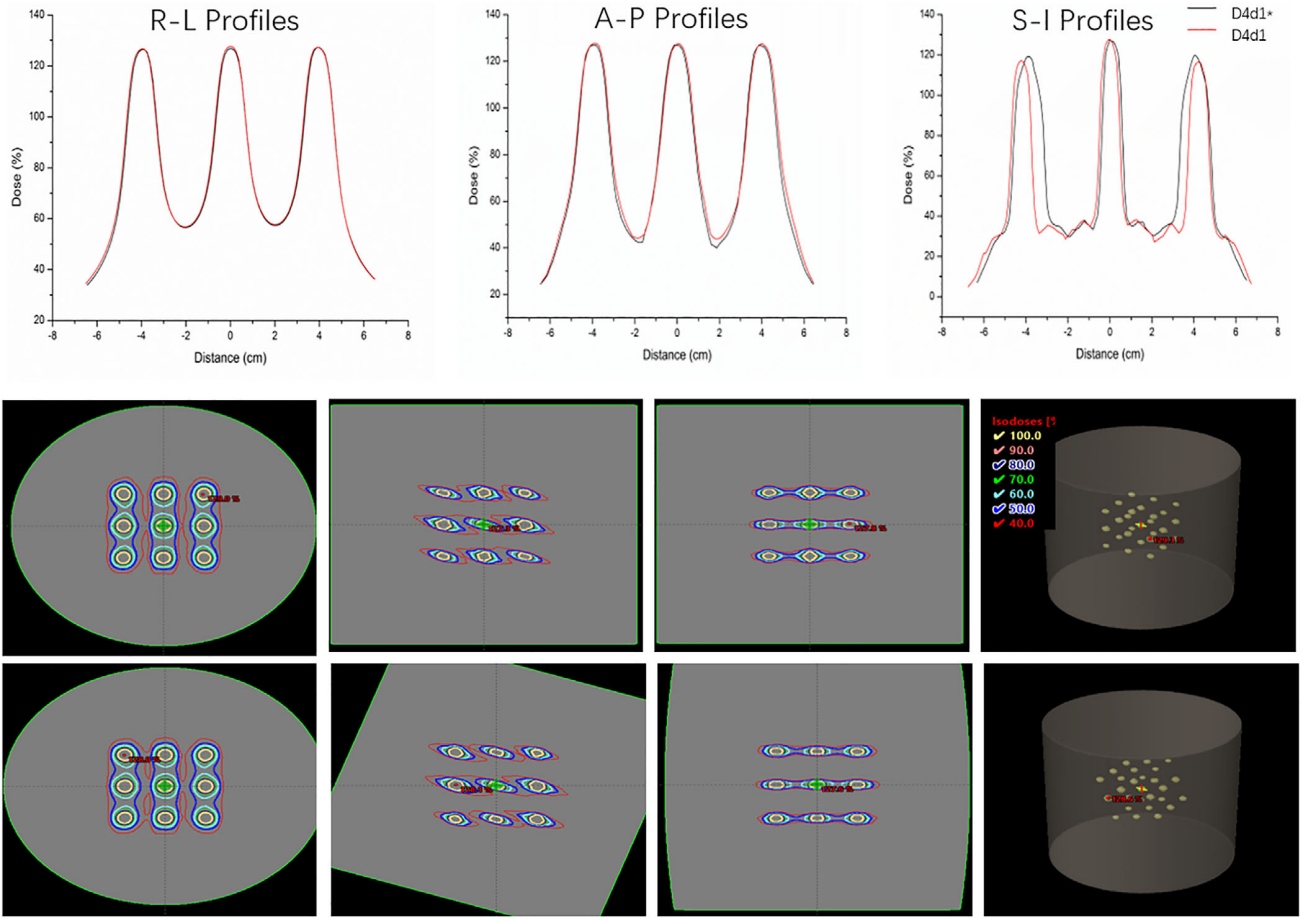
Dose distributions and peak‐to‐valley dose profiles for D4d1 and D4d1*. Top: Profiles in three different orthogonal directions, RL (left), AP (middle), and SI (right). Middle: Dose distribution for D4d1. Bottom: Dose distribution for D4d1*.

Figure S4 shows dose distributions and profiles for varying D at a fixed d (left) and varying d at a fixed D (right). The distributions indicate that D_5%Gap_ reliably reflects the valley dose between vertices. In the profiles, the valley dose consistently decreased with increasing D, with directional variations clearly shown in Figure 6 (top). Notably, the SI direction exhibited the lowest valley dose compared with the RL and AP directions. The 3D model of the 100% prescription isodose (yellow) revealed slightly lower dose coverage in off‑axis vertices than in central ones. Few differences in dose distributions and profiles were observed between the D4d1 and D4d1*.

### 3.2 Dosimetric evaluation in clinical examples

Two GIST patients were included. Patient #1 and Patient #2 had GTV volumes of 3241.9 cm^3^ and 3431.8 cm^3^, respectively. Both tumors were elongated, with the maximum dimension in the SI direction of 23.0 cm and 20.4 cm, respectively.

**FIGURE 7:**
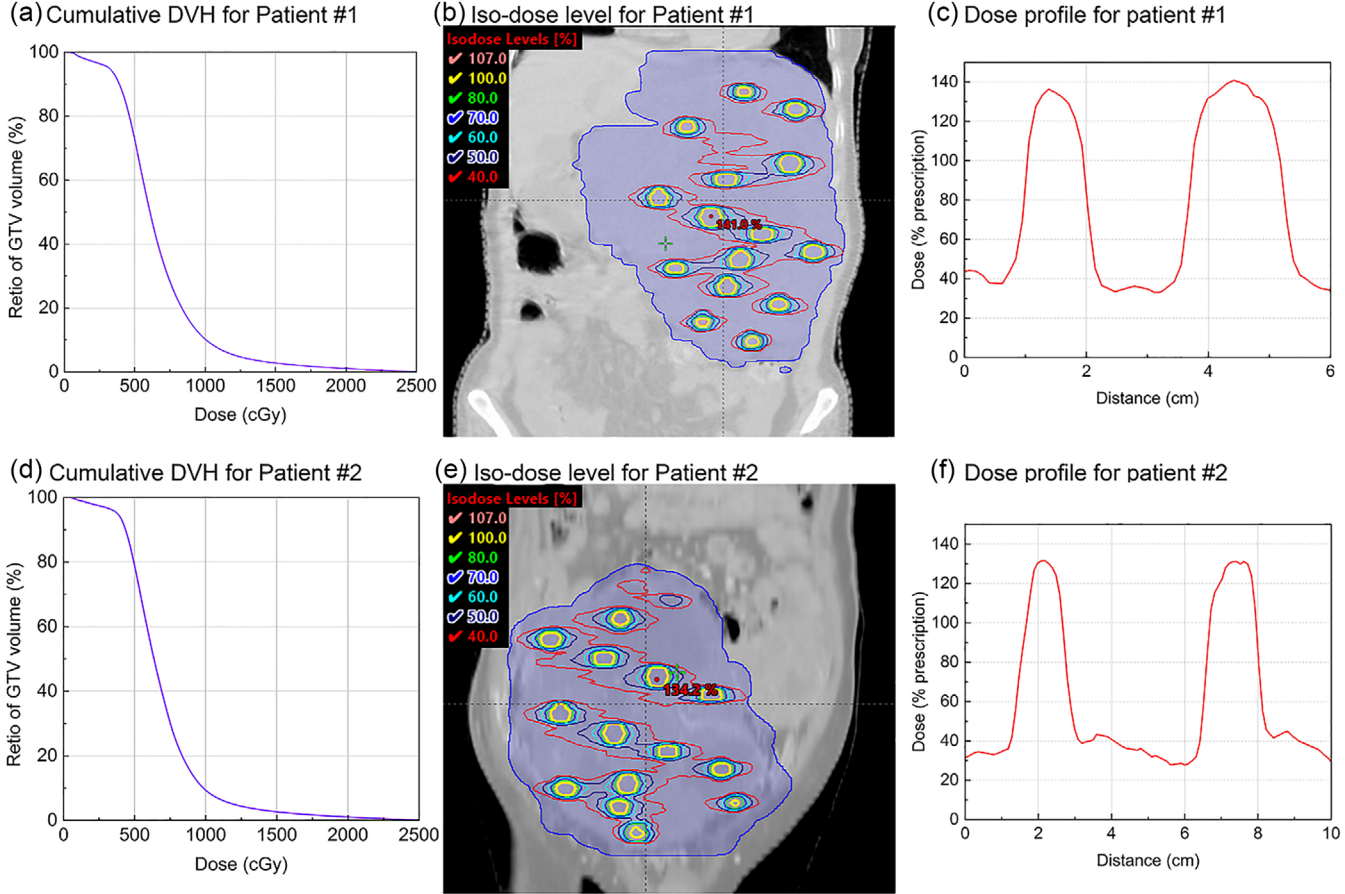
Clinical dosimetric characterization of patient plans: (a) dose volume histogram (DVH) for the gross tumor volume (GTV) corresponding to patient #1, (b) coronal dose distributions for patient #1, (c)dose profile across adjacent high‐dose regions within the GTV for patient #1. (d) DVH for the GTV corresponding to patient #2, (e) coronal dose distributions for patient #2, (f) corresponding dose profile for patient #2.

As shown in Figure 7, the GTV DVH curve, coronal dose distributions, and adjacent high‐dose regions dose profiles exhibited the characteristic highly inhomogeneous dose pattern of SFRT. The highest doses were strictly confined to the vertices, with rapid dose fall‐off outside. The long tail of the DVH curve reflects high doses delivered to small volumes, while the majority of the GTV received lower doses, indicative of the valley sparing between vertices.

Table 1 summarizes the detailed patient plan characteristics and compares them with the D4d1 in a water phantom. Both patient plans achieved the desired vertex coverage, with a consistent high‐dose volume proportion (V_Dp_/V_GTV_ ≈1.01%), slightly lower than that of D4d1(1.88%). The dose inhomogeneity parameters (Dp/D_95%Gap_ and D_meanV_/D_meanGap_) exceeded 3.8, ensuring robust biological differentiation between the high‐ and low‐dose regions, even higher than those of D4d1. The noncoplanar arrangement showed excellent deliverability: MCS > 0.68 (indicating simple MLC motion) and SAS < 6.06% (minimal small‑aperture reliance), though slightly inferior to D4d1. MU/cGy (6.78–7.25) was clinically acceptable and similar to that of D4d1. Table S2 lists the dosimetric parameters for patient OARs, All of which were well below the SBRT dose constraints.^30^, ^31^

**TABLE 1:** Patient plan characteristics and comparing with the D4d1 in water phantom.

Parameters ∖ Plans	Patient #1	Patient #2	D4d1
Site	abdomen	pelvic cavity	water phantom
V_GTV_ (cc)	3241.90	3431.80	1085.80
V_Dp_ (cc)	32.90	34.50	20.40
number of vertices	41	39	27
Contouring time(min)	45.7	43.2	3.5
Planning time(min)	10.6	11.1	5.8
EUD_GTV_ (Gy)	4.98	5.30	6.09
D_10%GTV_ (%)	50.30%	49.20%	61.12%
D_90%GTV_ (%)	19.50%	21.80%	25.05%
D_5%Gap_ (%)	46.60%	45.80%	51%
D_95%Gap_ (%)	14.60%	18.20%	23%
D_meanGap_ (%)	29.40%	30.7%	34%
D_maxNT_ (%)	53.20%	56.30%	62%
V_50%NT_ (cc)	0.03	0.16	6.85
D_meanLTV_ (%)	116.50%	117.80%	113.50%
V_Dp_/V_GTV_	1.01%	1.01%	1.88%
VDR	2.58	2.26	2.44
Dp/D_95%Gap_	6.85	5.49	4.27
D_meanLTV_/D_meanGap_	3.96	3.84	3.31
MCS	0.68	0.74	0.77
SAS	6.06%	5.21%	2.64%
MU/cGy	6.78	7.25	6.97

*Note*: D4d1: D4d1 denotes the optimal non‐coplanar arrangement with vertex diameter(d) = 1 cm and center to center distance (D) = 4 cm in the water phantom.

Vertex contouring took substantially longer in patients than in the phantom (∼45 min vs. 4 min), whereas planning time was comparable between the two settings (∼10 min vs. 6 min). The total time for contouring and planning combined was approximately one hour per patient.

## 4 DISCUSSION

This study introduces a simple and practical approach for arranging non‐coplanar vertices in lattice radiation therapy. By integrating DCA technology into LRT planning, this method enables efficient generation of LRT treatment plans with precise dosimetry. The resulting plans demonstrated accuracy while potentially reducing technical barriers to clinical adoption of LRT.

Unlike prior LRT delivery methods relying on complex VMAT technique,^4^, ^14^, ^17^, ^32^ this study adopted the more convenient and efficient DCA technology. The biggest challenge for applying DCA to SFRT technique is that coplanar vertices on the same transverse plane hinder MLC conformity across gantry angles, elevating unintended valley doses between adjacent vertices. Non‐ coplanar plan with couch rotation can achieve better dose fall‐off^21^, ^33^ which is perfect for suppressing the valley dose between lattice vertices. However, its application is often limited for many thoracic and abdominal tumors. Therefore, we developed an alternative non‐coplanar vertex arrangement strategy, separating vertices column‐wise. Each DCA field conforms to a single vertex column with rotation in coronal plane, ensuring MLC leaves target only one vertex. This strategy renders DCA feasible for SFRT, streamlining planning and delivery. Different from previous complex and coplanar vertices placement,^14^, ^26^ our arrangement transforms the vertex arrangement in 3D to quasi‐2D, simplifying the process. Vertices are placed in superior‐inferior columns with adequate spacing, enabling MLC blocks between vertices Figure 2c. The rotation angle is determined by the number (N) of vertices on the same rotated slice per group: arctan(1/N). DCA‑based SFRT has traditionally been applied to small‑volume tumors.^34^ In this study, we demonstrated that the proposed non‐coplanar vertex arrangement enables the use of DCA for LRT in large tumors, without requiring additional modeling or re‑optimization.

In this study, V_Dp_/V_GTV_ was consistently above 1% across all evaluated plans, within the recommended range of 1%–10%.^1^, ^9^ V_Dp_/V_GTV_ reflects the actual high‑dose volume proportion delivered to the GTV, whereas V_LTV_/V_GTV_ only the nominal volume ratio of the contoured lattice structures. The lower clinical value (1.01% vs. 1.88% in phantom) reflects geometric differences: the phantom had a regular GTV (achieved by 4‑cm expansion followed by 3‑cm inward contraction) allowing perfect nesting, whereas patient GTVs were irregular with recesses that prevented an exact geometric match with the internally placed LTVs, leaving some peripheral areas unable to accommodate the high‑dose vertices. All patient V_Dp_/V_GTV_ (1.01%) were similar to the 0.98% reported in a previous study^17^ with comparable parameters (1.5‑cm diameter, equivalent to our 1.0‑cm diameter, and 4‑cm center‑to‑center distance). Dp/D_95%Gap_ and D_meanLTV_/D_meanGap_ increased with D, unlike PVDR and VDR, indicating they better reflect the influence of spatial layout on dose heterogeneity. The higher dose Heterogeneity ratio in patients (vs. phantom) was mainly due to the lower high‑dose proportion, which markedly reduced valley doses: D_meanGap_ dropped from 34% to < 31%, and D_95%Gap_ from 23% to < 18.2%. EUD_GTV_ in patients (∼5 Gy) was lower than in phantom (∼6 Gy) but higher than Gaudreault et al.^17^ (∼4 Gy). Notably, the plan with higher EUD also exhibited a larger high‑dose volume but a lower heterogeneity. Thus, a balance between EUD_GTV_ and heterogeneity is crucial in SFRT design. Both the NT doses and OAR doses in all plans were substantially below the tolerance limits typically required for SBRT, suggesting that the proposed DCA‑based LRT approach did not substantially increase the dose burden to normal tissues in our patient cases.

PVDR of profile varied with direction; SI showed the lowest valley dose, consistent with Lee et al.^32^ MLC leaf width limited the minimum vertex diameter to 1 cm. In the water phantom, D3.5d1 optimized dose heterogeneity, while D4d1 balanced heterogeneity and valley dose. Our results align with Lee et al. for similar geometry (1.0‑cm diameter, 3.0‑cm edge‑to‑edge spacing ≈ our 4.0‑cm center‑to‑center): LR‑PVDR was 3.00 vs. 2.97. Larger diameters are feasible but may be gradient‑limited to keep a sufficiently low valley dose. The 6‑MV FFF beam gave slightly lower off‑axis vertex doses than central ones. The central‑dominant dose distribution allows the high‑dose peaks to concentrate in the core of the tumor, while the dose naturally falls off toward the tumor periphery. This peripheral dose gradient helps minimize exposure to adjacent OARs without compromising the biological effectiveness of LRT within the central tumor regions. At D = 5 cm, MLC speed could not track the farthest off‑axis vertices (gantry angles near 0°/180°), leaving them without dose coverage.

Proposed plans showed favorable complexity and deliverability. MCS exceeded that of previous study,^17^ indicating simpler MLC motion and larger apertures, which reduces delivery errors and improves robustness. SAS for sub‐10 mm apertures was below 10% across all plans, confirming minimal small‐field reliance and high dosimetric accuracy.^29^ MU/cGy values matched those of conventional VMAT,^4^, ^32^ without compromising efficiency.

Noncoplanar vertex plan and coplanar vertex plan with couch rotation had nearly identical dosimetric parameters, indicating that the noncoplanar arrangement mimics couch rotation and is a viable alternative when rotation is limited. The method is best suited for relatively large, centrally located tumors. For cases requiring SIB, DCA can serve as a base plan supplemented by VMAT optimization, extending its clinical applicability. The overall workflow, including both vertex arrangement and plan adjustment, required approximately one hour per patient—a duration that remains clinically practical and compares favorably with many other complex planning techniques. The DCA‑based LRT approach delivers the prescription dose to discrete vertices rather than to the entire GTV. Therefore, GTV irregularity does not substantially affect dose conformity; instead, it mainly influences the time required for vertex arrangement (∼45 min in patient plans vs. 4 min in the water phantom).

A key strength of this study is the integration of 3D non‐coplanar vertex arrangement with DCA for LRT planning. This approach simplifies vertex placement by reducing the 3D layout to a quasi‑2D geometry via coronal rotation, enabling consistent MLC blocking across the full 360° gantry rotation without couch rotation. The DCA‐based planning shortens overall planning time and reduces plan complexity. Importantly, the entire workflow can be executed within the standard TPS without any additional scripts, GPU acceleration, or external tools, lowering the technical barrier and facilitating adoption in resource‐limited centers.

Several limitations should be acknowledged. This study used only two patient CT scans for dosimetric comparison and did not include actual treatment delivery; thus, the findings are proof‑of‑concept dosimetric evaluations, not clinical validation. Larger prospective studies with delivery are needed. DCA alone does not support simultaneous integrated boost (SIB) in LRT, but can serve as a base plan to achieve acceptable SIB plans. Additionally, a fixed vertex geometry was used for one LTV, which may not be optimal for irregular targets; future work will optimize this geometry. Although manual vertex placement remains the most time‑consuming step in the current workflow, the total planning time per patient was approximately one hour, which is considered clinically acceptable at our institution. Future optimization with automated vertex generation could further streamline the process.

## 5 CONCLUSION

This study validates the feasibility of DCA‐based LRT planning with non‐coplanar vertex arrangement using medical linear accelerators. The proposed method facilitates easy and practical generation of LRT treatment plans and offers an alternative for patients with couch rotation limit. Dosimetric characteristics in the ideal phantom showed that the optimal combination is a 1 cm vertex diameter with a 3.5–4 cm center‐to‐center distance, which achieves a good balance between dose heterogeneity and low valley dose. The clinical feasibility of this approach was further corroborated by the two patient cases included in this study, where the planned dosimetric parameters met the predefined LRT criteria, with a total planning time of approximately one hour per patient, demonstrating that the method is not only theoretically optimal in phantoms but also practically applicable in real clinical scenarios with complex anatomies.

## AUTHOR CONTRIBUTIONS

Qingfeng Jiang and Haixia Cui: drafting of work, analysis and interpretation of trials and literature, drafting of manuscript, and manuscript review. Wenli Lu prepared the figure and contributed to the revision of the literature. Hai‐xia Cui, Wei Huang and Xin Yi collected the data, reviewed the literature, and wrote the article.

## CONFLICT OF INTEREST STATEMENT

The authors declare no conflicts of interest.

## ETHICAL APPROVAL

This study was approved by the Ethics Committee of The First Affiliated Hospital of Chongqing Medical University (Chongqing, China) (the official approval letter with institutional stamp is available upon request). Patient data were fully anonymized prior to use.

## Supporting information




**Supporting Information**: acm270818‐sup‐0001‐Figure S1‐S4.docx

**Supporting Information**: acm270818‐sup‐0002‐Table S1‐S2.docx

## Data Availability

Authors will share data upon request to the corresponding author.
