# Supplementary material for: Feasibility of lattice radiation therapy using a noncoplanar vertex arrangement and dynamic conformal arcs
Source: J Appl Clin Med Phys. 2026 Sep 23;27(10):e70818. doi: 10.1002/acm2.70818 (PMC13598931; doi:10.1002/acm2.70818)
Supplement: Supplementary file 1 [file ACM2-27-e70818-s001.docx]

**Supplementary material**

Table S1: summary of various critical Dose-volume parameters of different plans.

| Plans\ parameters | V_LTV_/V_GTV_ | V_Dp_/V_GTV_ | PVDR | VDR | Dp/  D_95%Gap_ | D_meanLTV_/  D_meanGap_ | D_2ccGTV_/  D_95%Gap_ | D_meanGTV_  (%) | EUD_GTV_  (Gy) | D_5%Gap_  (%) | MCS | SAS  (%) | MU/cGy |
| --- | --- | --- | --- | --- | --- | --- | --- | --- | --- | --- | --- | --- | --- |
| D3d1 | 2.10% | 6.18% | 30.35 | 2.46 | 3.05 | 2.87 | 4.42 | 57.30% | 6.78 | 68.52% | 0.79 | 2.55 | 8.1 |
| D3.5d1 | 1.56% | 2.66% | 28.27 | 2.66 | 4.25 | 3.16 | 5.36 | 44.40% | 6.10 | 56.42% | 0.78 | 4.24 | 7.22 |
| D4d1* | 1.23% | 1.78% | 31.54 | 2.41 | 4.24 | 3.26 | 5.09 | 40.50% | 6.06 | 50.55% | 0.78 | 2.56 | 7.15 |
| D4d1 | 1.20% | 1.88% | 26.24 | 2.44 | 4.27 | 3.31 | 5.20 | 40.70% | 6.09 | 51% | 0.77 | 2.64 | 6.97 |
| D5d1 | 0.78% | 1.22% | 31.23 | 2.68 | 5.52 | 4.08 | 6.87 | 32.90% | 5.07 | 45.11% | 0.73 | 7.91 | 7.51 |
| D3.5d1.4 | 3.24% | 5.59% | 23.93 | 2.76 | 3.55 | 2.76 | 4.73 | 54.00% | 6.62 | 66.73% | 0.76 | 5.26 | 6.71 |
| D4d1.4 | 2.47% | 3.84% | 22.22 | 2.53 | 3.62 | 2.78 | 4.52 | 49.30% | 6.76 | 60.77% | 0.76 | 2.33 | 6.39 |

D4d1: the plan for the 3×3×3 LTV D4d1 without couch rotation

D4d1*: the plan for the 3×3×3 LTV D4d1* with 19° couch rotation

Table S2 Summary of the OARs dosimetry parameters for each patient

| OAR parameters \ Plans | | Patient #1 | Patient #2 |
| --- | --- | --- | --- |
| Kidney L | Dmax | 6.46 |  |
|  | V9.5Gy | 0 |  |
| Kidney R | Dmax | 3.59 |  |
|  | V9.5Gy | 0 |  |
| Liver | Dmax | 5.21 |  |
|  | V<11.6Gy | 754.6cc |  |
| spinal cord | Dmax | 4.72 |  |
|  | V10Gy | 0 |  |
| Bowel bag | Dmax | 8.58 | 9.22 |
|  | V17.6Gy | 0 | 0 |
| Colon | Dmax | 8.35 | 7.4 |
|  | V20.5Gy | 0 | 0 |
| Bladder wall | Dmax |  | 9.25 |
|  | V12Gy |  | 0 |
| Rectum | Dmax |  | 3.51 |
|  | V30Gy |  | 0 |
|  | V23Gy |  | 0 |
| Femoral L | Dmax |  | 3.33 |
|  | V15Gy |  | 0 |
| Femoral R | Dmax |  | 4.4 |
| Caudal equina | Dmax |  | 0 |
|  | V15Gy |  | 4.02 |
