# Supplementary material for: Feasibility of lattice radiation therapy using a noncoplanar vertex arrangement and dynamic conformal arcs
Source: J Appl Clin Med Phys. 2026 Sep 23;27(10):e70818. doi: 10.1002/acm2.70818 (PMC13598931; doi:10.1002/acm2.70818)
Supplement: Supplementary file 2 [file ACM2-27-e70818-s002.docx]

**Supplementary materials**

|  |  |
| --- | --- |

Fig. S1 GTV mean dose (Dmean) (% prescription) and GTV EUD (Gy) in this study.

EUD, equivalent uniform dose.

*denotes the plan for the 3×3×3 LTV D4d1* with 19° couch rotation.

Fig. S2 Gap mean dose (Dmean) and Gap dose metrics (%prescription) for each LTV in this study.

*denotes the plan for the 3×3×3 LTV D4d1* with 19° couch rotation.

Fig. S3 NT max dose (%prescription) (top), and NT V50% (cm^3^) (bottom) for each LTV considered in this study.

*denotes the plan for the 3×3×3 LTV D4d1* with 19° couch rotation.

Fig. S4 Dose distribution as the center-to-center distance (D) was varied (left) and as the vertex size(d) was varied (right)
